# Methods for quick, accurate and cost-effective determination of the type 1 diabetes genetic risk score (T1D-GRS)

**DOI:** 10.1515/cclm-2019-0787

**Published:** 2020-03-26

**Authors:** Jonathan M. Locke, Mark J. Latten, Renu Y. Datta, Andrew R. Wood, Martin A. Crockard, John V. Lamont, Michael N. Weedon, Richard A. Oram

**Affiliations:** Institute of Biomedical and Clinical Science, University of Exeter Medical School, Exeter, Devon, UK; Randox Laboratories Ltd., Crumlin, County Antrim, UK; Institute of Biomedical and Clinical Science, University of Exeter Medical School, Level 3, RILD Building, Barrack Road, Exeter, Devon, EX2 5DW, UK, Phone: 01392 408538, Fax: 01392 408388

**Keywords:** genotyping, SNP, type 1 diabetes

To the Editor,

Genetic risk scores, which sum the risk attributed to multiple independent single nucleotide polymorphisms (SNPs) across the genome, have the potential to be used in a range of clinical scenarios and studies (e.g. diagnosis, prognosis and intervention). For example, we and others have shown that genotyping just 10 SNPs generates a type 1 diabetes genetic risk score (T1D-GRS) effective in discriminating type 1 diabetes from type 2 diabetes and predicting disease progression [1], [2].

A number of studies have calculated the T1D-GRS using data derived from high-density SNP arrays (e.g. [1], [2]). Whilst SNP arrays are becoming increasingly affordable there are disadvantages to using such genome-wide methods. The surfeit of genomic information, the majority of which is not needed for T1D-GRS calculation, can pose ethical and legal obstacles, and a relatively high level of expertise is needed to analyse and interpret SNP array data.

We set out to determine whether a 10 SNP T1D-GRS (Table 1) can be accurately (genotype accuracy) and efficiently (genotype call-rate) calculated using focussed genetic profiling methods. As our gold-standard method we genotyped the T1D-GRS in 300 DNA samples (58% non-Finnish White Europeans, 40% Asian) using Sanger sequencing. The 300 samples represented a continuous series with no other selection criteria. All individuals had consented to genetic testing for monogenic diabetes with determination of the T1D-GRS part of the diagnostic pipeline for individuals referred to Exeter Clinical Laboratory. Further ethical approval for this study was not required as samples were anonymised to the researchers in this study with these investigations carried out in accordance to the tenets of the Declaration of Helsinki. Following Sanger sequencing samples were subsequently tested using KASP^™^ genotyping assays (LGC Group) and a T1D-GRS biochip array developed by Randox Laboratories Ltd.

**Table 1:** List of SNPs used to generate the 10 SNP T1D-GRS.

SNP	Gene	Odds ratio	Weight	Effect allele
rs2187668, rs7454108	*DR3*/*DR4-DQ8*	48.18	3.87	
	*DR3*/*DR3*	21.12	3.05	
	*DR4-DQ8*/*DR4-DQ8*	21.98	3.09	
	*DR4-DQ8/X*	7.03	1.95	
	*DR3/X*	4.53	1.51	
rs1264813	*HLA_A_24*	1.54	0.43	T
rs2395029	*HLA_B_5701*	2.5	0.92	T
rs3129889	*HLA_DRB1_15*	14.88	2.7	A
rs2476601	*PTPN22*	1.96	0.67	A
rs689	*INS*	1.75	0.56	T
rs12722495	*IL2RA*	1.58	0.46	T
rs2292239	*ERBB3*	1.35	0.3	T
rs10509540	*C10orf59*	1.33	0.29	T

Effect allele is the risk increasing allele on the positive strand.

For the KASP assays (previously detailed in [3]) a Biomek NX58 (Beckman Coulter) automated liquid handler was used to set up reactions (4 μL) in 384-well PCR plates prior to thermocycling and fluorescent reading on the QuantStudio 12K Flex Real-Time PCR system (Thermo Fisher). Genotypes were called using QuantStudio 12K Flex Software v1.2.2 (Thermo Fisher). To ensure accurate genotype clustering each plate contained three controls for each genotype and for each SNP (90 controls total). For the Randox biochip array method (described in [4]) we followed the manufacturer’s instructions. Briefly, target DNA was amplified through allele specific, single-tube multiplex PCR, followed by spatial hybridisation onto a biochip array, conjugation and chemiluminescence detection using an Evidence Investigator analyser (Randox).

Using the KASP method 99.5% (2984/3000) of reactions resulted in an assigned genotype, with an initial agreement with Sanger sequencing of 99.9% (2981/2984). On repeat of the KASP assay the three discordant genotypes were no longer discordant. Using the Randox biochip method 99.3% (2980/3000) of reactions resulted in an assigned genotype and 99.97% (2979/2980) were concordant with genotypes determined by Sanger sequencing. The discordant genotype for rs3129889, which tags HLA-DRB1*1501, remained so on repeat and was shown to be caused by a rare SNP (rs3763326) resulting in allelic dropout. Since this finding new primers have been designed to genotype rs3129889 which do not overlap rs3763326. Furthermore, all primers used for the Randox biochip have subsequently been checked for SNPs using gnomAD v2.1.1 [5] and we can confirm there are no SNPs under any of the primers with a global minor allele frequency >1%.

The advantages and disadvantages of each technique are presented in Table 2 and briefly discussed herein. Sanger sequencing is widely used and established in genetics laboratories; however, it is a multi-step process and relatively costly per reaction. Whilst not as quick as the KASP and Randox biochip methods Sanger sequencing is still significantly quicker than genotyping by microarray which has a minimum time of several days and requires batched analysis and quality control. The KASP method involved minimal hands-on time and when samples are batched is cheap in comparison to the other two methods. Furthermore, genotyping by KASP assays involves end-point fluorescent reading that can be quantified using a variety of plate readers which may aid widespread adoption. However, there is a requirement to run multiple control samples, unless historical genotyping data is used, and each assay is not CE-marked for diagnostic utility. On the other hand, Randox is CE-marking their T1D-GRS biochip which, whilst more costly than KASP, requires limited technical expertise and will, in its commercially available form, automatically provide a genotyping score without the need for data interpretation. Adoption of this method requires specific instrumentation, a Randox Evidence Investigator, whilst Sanger and KASP reactions can be conducted using a variety of different instruments. The cost and availability of instrumentation will be an important factor for each lab in the choice and uptake of these methods for T1D-GRS determination.

**Table 2:** Advantages and disadvantages of focussed genotyping methods for T1D-GRS determination.

	T1D-GRS genotyping method		
	PCR+Sanger sequencing	KASP assays	Randox biochip
Consumables cost (per T1D-GRS)	~£20	~£1	~£20
Roboticised set-up	Yes	Yes	No
Equipment accessibility	High	Medium	Medium
Speed of method (minimum)	~6 h	~3 h	~3 h
Requirement to genotype multiple controls/run	No	Yes^a^	No
Seeking CE-marking	No	No	Yes

^a^Unless historical genotyping data is used to generate clusters.

For many common diseases, genome-wide association studies have likely already identified the SNPs with the largest effects on disease risk. A small number of SNPs may thus be used for stratification, diagnostic and/or prognostic purposes, with this number amenable to PCR-based methods of amplification as used in this study. Validating that PCR-based methods are accurate and efficient is important not only for current protocols but also for future point-of-care (POC) testing. In the future it is likely that POC testing will utilise PCR technology, given the enzymes and protocols now available to genotype directly from blood (i.e. without pure DNA being extracted) (e.g. [6]).

To conclude, we have shown that focussed genotyping methods are available for quick, accurate and efficient determination of the T1D-GRS. The optimal choice of method will depend on cost, availability and need for CE/regulatory marking. This study showcases their potential utility in a variety of settings that necessitate quick, targeted genotyping of a small number of SNPs.
